# The Prevalence of HSV, HHV-6, HPV and *Mycoplasma genitalium* in *Chlamydia trachomatis* positive and *Chlamydia trachomatis* Negative Urogenital Samples among Young Women in Finland

**DOI:** 10.3390/pathogens8040276

**Published:** 2019-12-01

**Authors:** Suvi Korhonen, Kati Hokynar, Tiina Eriksson, Kari Natunen, Jorma Paavonen, Matti Lehtinen, Mirja Puolakkainen

**Affiliations:** 1 Department of Virology, University of Helsinki and Helsinki University Hospital, PO Box 21, FI-00014 Helsinki, Finland; kati.hokynar@helsinki.fi(K.H.); mirja.puolakkainen@helsinki.fi (M.P.); 2 Faculty of Social Sciences, Tampere University, PO Box 100, FI-33014 Tampere, Finland; tiina.eriksson@tuni.fi (T.E.); kari.natunen@tuni.fi (K.N.); matti.lehtinen@tuni.fi (M.L.); 3 Obstetrics and Gynecology, University of Helsinki and Helsinki University Hospital, PO Box 140, FI-00029 HUS Helsinki, Finland; jorma.paavonen@helsinki.fi; 4 Department of Laboratory Medicine, Karolinska Institute, Alfred Nobels Allé 8, 14183 Stockholm, Sweden

**Keywords:** sexually transmitted infection, chlamydial co-infection

## Abstract

*Chlamydia trachomatis*, *Mycoplasma genitalium*, herpes simplex virus (HSV) and human papillomavirus (HPV) cause sexually transmitted infections. In addition, human herpesvirus 6 (HHV-6) may be a genital co-pathogen. The prevalence rates of HSV, HHV-6, HPV, *M. genitalium*, and the *C. trachomatis ompA* genotypes were investigated by PCR in urogenital samples of the *C. trachomatis* nucleic acid amplification test positive (n = 157) and age-, community- and time-matched negative (n = 157) women. The prevalence of HPV DNA was significantly higher among the *C. trachomatis* positives than the *C. trachomatis* negatives (66% vs. 25%, *p* < 0.001). The prevalence of HSV (1.9% vs. 0%), HHV-6 (11% vs. 14%), and *M. genitalium* DNA (4.5% vs. 1.9%) was not significantly different between the *C. trachomatis*-positive and -negative women. Thirteen per cent of test-of-cure specimens tested positive for *C. trachomatis*. The prevalence of HSV, HHV-6, HPV, *M. genitalium*, and the *C. trachomatis ompA* genotypes did not significantly differ between those who cleared the *C. trachomatis* infection (n = 105) and those who did not (n = 16). The higher prevalence of HPV DNA among the *C. trachomatis* positives suggests greater sexual activity and increased risk for sexually transmitted pathogens.

## 1. Introduction

The global disease burden due to sexually transmitted infections (STIs) is substantial [1]. *Chlamydia trachomatis* urogenital infection is the most common sexually transmitted bacterial infection with 127 million cases annually [2]. Most chlamydial infections remain asymptomatic, but can result in pelvic inflammatory disease and severe reproductive complications [3]. Based on the *ompA* gene encoding the major outer membrane protein (MOMP), *C. trachomatis* can be classified into different genotypes, of which genotypes D-K cause most of the urogenital infections [4]. *Mycoplasma genitalium* infection is associated with non-gonococcal and non-chlamydial urethritis in men, and with urethritis, cervicitis, endometritis and pelvic inflammatory disease in women [5]. In addition, asymptomatic infections are frequent [5]. The increasing antimicrobial resistance of *M. genitalium* to macrolides and fluoroquinolones causes concern [6].

Genital herpes caused by herpes simplex virus (HSV-2 or HSV-1) affects over 500 million people worldwide [7,8]. After initial infection, herpesviruses establish lifelong latency in which the viral gene expression is limited. Upon reactivation, infectious viral particles and viral DNA are shed, and clinical disease can recur [9]. Human herpesvirus 6 (HHV-6) now includes two different viruses, HHV-6A and HHV-6B, with different epidemiological and biological characteristics and disease associations [10]. While less is known about the epidemiology of HHV-6A, HHV-6B is a ubiquitous virus and the seroprevalence of HHV-6 among adults is approximately 98% [11]. HHV-6B infection usually occurs early in childhood and can cause fever and rash (exanthema subitum), but HHV-6 infections including primary infections and reactivation can be asymptomatic [12]. Some individuals (less than 1%) have HHV-6 DNA integrated into telomeric ends of their chromosomes which could result in PCR positivity [12]. HHV-6 is known to be transmitted vertically, through saliva [12], and in addition, sexual transmission is possible [13]. Genital human papillomavirus (HPV) infections are common and the disease burden associated with HPV is enormous. The prevalence in women with normal cervical cytology is approximately 12% [14]. Many HPV infections clear spontaneously, but infections caused by the high-risk HPV genotypes can lead to cervical cancer, one of the leading cancers in women worldwide [15].

In Finland, *C. trachomatis* infections are notified by laboratories to the Finnish Infectious Disease Register maintained by the Finnish Institute for Health and Welfare since 1995. Genital HSV, HHV-6, HPV and *M. genitalium* infections are not notifiable and their prevalence is not known. Here, we wanted to study the prevalence of HSV, HHV-6, HPV and *M. genitalium* among *C. trachomatis* nucleic acid amplification test (NAAT) positive, and age-, community- and time-matched *C. trachomatis* NAAT-negative young women participating in a community-randomized trial on the effectiveness of *C. trachomatis* screening in Finland [16]. As genital coinfections can play a role in development of cancer (HPV) [17,18] or drive *C. trachomatis* into a persistent state (herpesviruses) [19,20], we studied whether the co-infections affect clearance of *C. trachomatis*.

## 2. Results

### 2.1. The Prevalence of HSV, HHV-6, HPV and M. genitalium

The prevalence of HSV, HHV-6, HPV and *M. genitalium* DNA was analysed in the urogenital samples of 314 young women (157 *C. trachomatis* NAAT positive individuals and their 157 age-, community- and time-matched *C. trachomatis* NAAT negative controls). Among young women, HPV DNA was frequently (46%) detected and it was more common among the *C. trachomatis* positives than the *C. trachomatis* negatives (66% vs. 25%, *p* < 0.001) (Table 1). HHV-6 DNA was present in 12%, *M. genitalium* DNA in 3.2% and HSV DNA in 1.0% of the samples. Of the ten samples positive for *M. genitalium* DNA, two samples (20%) contained a mutation associated with macrolide resistance. A slightly higher prevalence of *M. genitalium* and HSV DNA in the *C. trachomatis*-positive samples was observed, but due to the small numbers, these differences were not statistically significant. Among the 314 samples, 22 (21 among *C. trachomatis* NAAT positive and one among NAAT negative) were positive for three of the microbes analysed in this study. Only one sample tested positive for *Neisseria gonorrhoeae*.

Among the 157 *C. trachomatis*-positive women, 166 infection episodes were evaluated (nine women had two *C. trachomatis* infection episodes during the study period). A test-of-cure (TOC) sample for *C. trachomatis* detection was taken approximately one month after the treatment as recommended by the Finnish national guideline [21], and it was available from 121 (73%) cases. The infection episodes were classified into not cleared (*C. trachomatis* TOC-positive) and cleared (*C. trachomatis* TOC-negative). Of the 121 TOC specimens, sixteen (13%) tested positive for *C. trachomatis*. The prevalence of HSV, HHV-6, HPV and *M. genitalium* DNA was similar between those who cleared *C. trachomatis* infection and those who did not (Table 2). HHV-6 DNA seemed more frequent among those who gave a *C. trachomatis* positive TOC sample (19% vs. 10%). However, the difference was not significant. 

### 2.2. The C. trachomatis ompA Genotypes

Of the *C. trachomatis* NAAT-positive urogenital samples from 166 infection episodes, 146 (88%) samples could be genotyped by *ompA* PCR. The three most prevalent *C. trachomatis ompA* genotypes were E (n = 69, 47%), F (n = 39, 27%) and G (n = 13, 9%) (Table 3). No statistically significant differences were observed in the genotype distribution between those who cleared the infection and those who did not.

## 3. Discussion

In this study, we explored the prevalence rates of HSV, HHV-6, HPV and *M. genitalium* by PCR among young women (18 and 22 years old) participating in a community-randomized *C. trachomatis* screening trial in Finland. HPV DNA was significantly more frequently detected among the *C. trachomatis* positives than the *C. trachomatis* negatives (66% vs. 25%). This finding is in an agreement with results of a large Italian study showing an HPV co-infection in 68% of *C. trachomatis*-positive ≤25-year-old women [22]. Globally, there is a relationship between HPV prevalence and age as the highest rates are observed in younger women of less than 25 years old, with a decline at older ages [14]. The high HPV prevalence suggests greater sexual activity and exposure to sexually transmissible microbes among those positive for *C. trachomatis*. This is of importance, as concomitant *C. trachomatis* and HPV infection is associated with high risk of cervical precancer [23], supporting the active role of *C. trachomatis* in the development of cervical precancer [17,18].

In contrast to HPV, the prevalence of HSV, HHV-6 and *M. genitalium* did not significantly differ among the *C. trachomatis*-positive and -negative young women. In this study, HSV DNA, indicating most likely asymptomatic shedding and including both HSV-1 and HSV-2, was rarely detected in the urogenital specimens (1%) among young women. This is in line with the previous Finnish data: genital herpes was clinically diagnosed in 3% of women attending twelve outpatient clinics in 1995–2006 [24], and approximately 2% of the STI clinic patients were culture-positive for HSV in 1994–2012 [25,26]. Among Finnish pregnant women, the seroprevalence for HSV-1 was 45% and for HSV-2 was 11% in 2012 [27]. The global estimates for genital HSV infections suggest higher prevalence: 0.8% (15–19 years old) and 2% (20–24 years old) for HSV1 [7], and 4.6% (15–19 years old) and 7.8% (20–24 years old) for HSV2 [8]. In our study, HHV-6 DNA was detected in 12% of the specimens. Similarly, an HHV-6 prevalence of up to 10% was reported in the genital tract of non-pregnant women in the USA [13], whereas up to 26% of Japanese pregnant women were shown to have HHV-6 DNA in their genital tract [28]. As HHV-6 induces a lifelong latent infection in humans and is capable of reactivation, either reactivation or (re)infection can explain HHV-6 DNA positivity. 

The global estimate for the prevalence of *M. genitalium* is 1.7% in under 25-year-old women [29], that is slightly lower than the global prevalence of 3.8% for *C. trachomatis* in 15–49-year-old-women [2]. In our study, *M. genitalium* DNA was detected in 3.2% of the specimens, approximately as often as *C. trachomatis* [16]. Earlier, a somewhat higher prevalence of 5.6% for *M. genitalium* was reported among STI clinic patients in Finland [30]. In the other Nordic countries, the prevalence of 9.8% in Sweden, 4.9% in Norway and 9.0% in Denmark has been reported for *M. genitalium* [31]. In Norway, the prevalence of *M. genitalium* is suggested to be lower due to enhanced testing, treatment and partner notification [31]. In this study, two samples (20%) contained mutations in the 23S rRNA gene, leading to macrolide resistance in *M. genitalium*. Previously, such mutations were detected in 31% of *M. genitalium*-positive samples among STI clinic patients in Finland [30]. In the other Nordic countries, the prevalence of *M. genitalium* macrolide resistance is 18% in Sweden, 56% in Norway and 57% in Denmark [31]. This obviously reflects differences in the national treatment guidelines, as doxycycline is recommended in Sweden and azithromycin in Norway and Denmark in the treatment of *C. trachomatis* infection. 

In addition, we studied whether co-infections affect clearance of *C. trachomatis*. The prevalence of HSV, HHV-6, HPV and *M. genitalium* did not significantly differ between those who cleared the *C. trachomatis* infection and those who did not, although in vitro studies suggest that herpesviruses and *C. trachomatis* might interact during human infection. Indeed, it has been shown that HSV and HHV-6 co-infections promote *C. trachomatis* persistence in vitro [19,20], and persistent forms of *C. trachomatis* are more resistant to antimicrobial therapy [32]. As 13% of TOC samples were positive for *C. trachomatis*, the impact of co-infections, especially those due to herpesviruses, warrants further studies. 

Overall, the *ompA* genotype distribution in Finland has remained fairly stable during the last ten years, with E, F and G representing the most predominant genotypes [33,34]. Here, samples collected from asymptomatic women during a screening trial were studied, whereas the earlier studies included mainly samples from symptomatic patients. Previous studies have attempted to link certain *C. trachomatis ompA* genotypes to different clinical outcomes of the infection [35]. In this study, the *ompA* genotype distribution was similar among those who cleared the infection and those who did not suggesting that the *C. trachomatis ompA* genotype is not a decisive factor in the clearance of infection. Multiple factors, including host immunity [36] and infectious load [37] probably play a role in clearance. 

This was the first study to explore the prevalence rates of HSV and HHV-6 DNA in urogenital samples among young women in Finland. Although the study was rather small, the participants and their controls were carefully matched to avoid confounding factors related to age, local epidemiology of these agents, and sampling time. The samples were collected from females volunteering in an HPV vaccination trial and simultaneously in a community-randomized *C. trachomatis* screening trial and the risk-taking behaviour among the participants may have been higher. Due to this, the true population-based prevalence rates and the prevalence of *C. trachomatis* and HPV co-infection might be lower than the ones reported here. 

The interactions and consequences of co-infections with *C. trachomatis*, *M. genitalium*, HSV, HHV-6 and HPV can be numerous but are not well studied. In addition, STIs increase the risk of human immunodeficiency virus (HIV) acquisition and transmission [38]. Thus, it is important to understand the local and global epidemiology of the sexually transmitted microbes and also co-infections. This information can be used to prevent and control infections, to investigate the impact of screening interventions, and to develop guidelines for management. In addition, prevalence data can be used for monitoring purposes if drug resistance promotes further spread of infections. Ultimately, the development of vaccines against these sexually transmitted agents will result in permanent decline of the prevalence rates, as has already been observed with the implementation of HPV vaccination programs [39].

## 4. Materials and Methods 

### 4.1. Participants and Samples

The samples studied here represent a subset of samples collected from women participating in an HPV vaccination trial [40,41] and simultaneously in a community-randomized trial on the effectiveness of *C. trachomatis* screening [16] at the age of 18 and 22 in Finland in 2010–2017. The self-collected vaginal swabs rinsed in first-void urine were sent to Fimlab, Tampere, Finland for *C. trachomatis* and *N. gonorrhoeae* testing with the Abbott RealTime CT/NG assay (Abbott Molecular, Des Plaines, IL, USA). All women with a *C. trachomatis*-positive sample and their partners received free-of-cost treatment (1 g single dose azithromycin) and were invited to give a test-of-cure sample one month later. The samples had previously been analysed for HPV DNA with methods described earlier at the Department of Clinical Microbiology, Skåne University Hospital, Malmö, Sweden [42,43].

The study material consisted of urogenital samples from 314 women (157 *C. trachomatis* NAAT positive individuals, and their 157 age-, community- and time-matched *C. trachomatis* NAAT negative controls). Altogether 166 *C. trachomatis* infection episodes were analysed among the 157 women. A test-of-cure sample was available from 121 (73%) women. These women were classified as *C. trachomatis* test-of-cure positive (infection not cleared) (n = 16) or *C. trachomatis* test-of-cure negative (infection cleared) (n = 105). A test-of-cure sample was not available from 45 women (27%). All participants gave their informed consent for inclusion before they participated in the *C. trachomatis* screening trial. The study was conducted in concordance with the Declaration of Helsinki and an ethics approval for the *C. trachomatis* screening trial was obtained from the Ethical Review Board of the North Ostrobothnia Hospital District (19.11.2012; §295/2012).

### 4.2. DNA Extraction and Real-Time PCR

DNA was extracted from the urogenital samples with the MagNA Pure LC 2.0 System (Roche Molecular Systems, Pleasanton, CA, USA) using a MagNA Pure LC DNA Isolation Kit - Large Volume (Roche Molecular Systems). 

HSV (HSV-1 and HSV-2 DNA polymerase gene) [44], HHV-6 (HHV-6A and HHV-6B U67 gene) [45] and *M. genitalium* (*mgbB* gene) [46] were detected with real-time PCRs developed earlier with modifications described below. The samples positive for *M. genitalium* DNA were analysed for mutations associated with macrolide resistance within the 23S rRNA gene with a method developed previously [47]. To evaluate the specimen quality (i.e., the presence of PCR inhibitors) and the performance of the nucleic acid extraction, the human beta-globin gene was amplified [47]. The *ompA* genotype of the *C. trachomatis* positive samples was determined with a method described earlier [48] with modifications [33].

The primers and probes were purchased from Integrated DNA Technologies (IDT, Coralville, IA, USA) and Applied Biosystems (Thermo Fisher Scientific, Waltham, MA, USA). The real-time PCRs were performed in a 25 μL volume containing 12.5 μL Maxima Probe/ROX qPCR Master Mix (Thermo Fisher Scientific). The PCR for HSV contained 400 nM primers and 160 nM probes, for HHV-6 the PCR contained 100 nM forward primers, 600 nM reverse primers and 200 nM probes, for *M. genitalium* the PCR contained 100 nM primers and 100 nM probes, for *M. genitalium* macrolide resistance the PCR contained 50 nM of forward primers, 400 nM of reverse primers and 150 nM of probes, and for beta-globin the PCR contained 200 nM primers and 100 nM probes. All the probes included 6-FAM as a reporter dye and the HSV probe included a ZEN/Iowa Black FQ double-quencher.

The PCR analyses were performed on an ABI 7500 instrument and Sequence Detection Software version 1.3.1 (Applied Biosystems, Thermo Fisher Scientific). The thermal cycling conditions were two minutes at 50 °C, ten minutes at 95 °C, 45 cycles of 15 s at 95 °C and one minute at 60 °C. The template volume was 5 μL, and each sample was amplified in duplicate for HSV, *M. genitalium* and *C. trachomatis ompA* PCR, and in quadruplicate for HHV-6 PCR. The beta-globin gene PCR was performed in a single reaction and the gene was successfully amplified among all the samples analysed. No-template control was included in each run to assess for reagent contamination and it remained negative for all of the PCRs. As an amplification control for HSV, HHV-6 and *M. genitalium* PCR, the PCR target sequences in pIDTSMART-AMP plasmids were purchased from IDT. As an amplification control, DNA extracted from *C. trachomatis* reference strain types D to K (D:VR-885, E:VR-348B, F:VR-346, G:VR-878, H:VR-879, I:VR-880, J:VR-886, K:VR-887; American Type Culture Collection, Manassas, VA, USA) propagated in McCoy cells (from Pekka Saikku) was used in the *C. trachomatis ompA* PCR. 

### 4.3. Statistical Analysis

For comparison of the prevalence of HSV, HHV-6, HPV and *M. genitalium* DNA in *C. trachomatis* positives and negatives, the prevalence of HSV, HHV-6, HPV and *M. genitalium* DNA in *C. trachomatis* TOC positives and negatives, and the prevalence of *C. trachomatis ompA* genotypes D–K in *C. trachomatis* TOC positives and negatives, Fisher’s exact test was used. A *p*-value less than 0.05 was considered statistically significant. Statistical analyses were performed with the IBM SPSS Statistics v24 (IBM, Armonk, NY, USA). The 95% confidence intervals for the proportion of positives for HSV, HHV-6, HPV and *M. genitalium* were also calculated [49].

## Figures and Tables

**Table 1:** The prevalence (% and 95% confidence interval, CI) of HSV, HHV-6, HPV and *M. genitalium* DNA in the urogenital samples of 157 *C. trachomatis* nucleic acid amplification test (NAAT) positive and 157 age-, community- and time-matched *C. trachomatis* NAAT-negative women.

	*C. trachomatis* Positive (n = 157)	*C. trachomatis* Negative (n = 157)	*p*-Value ^1^	Total (n = 314)
HSV	3 (1.9%; 0–4.3%)	0 (0%)	0.248	3 (1.0%)
HHV-6	17 (11%; 5.6–16.0%)	22 (14%; 8.3–19.7%)	0.494	39 (12%)
HPV	104 (66%; 58.5–73.9%)	39 (25%; 17.7–31.9%)	<0.001	143 (46%)
*M. genitalium*	7 (4.5%; 1.0–8.0%)	3 (1.9%; 0–4.3%)	0.336	10 (3.2%)

HSV–herpes simplex virus; HHV-6–human herpes virus 6; HPV–human papillomavirus. ^1^ Fisher’s exact test was used to calculate the statistical significance.

**Table 2:** The prevalence (% and 95% confidence interval, CI) of HSV, HHV-6, HPV and *M. genitalium* DNA in the urogenital samples of women who later gave a *C. trachomatis* test-of-cure positive (n = 16) and a *C. trachomatis* test-of-cure negative (n = 105) sample.

	*C. trachomatis* Test-of-Cure Positive (n = 16)	*C. trachomatis* Test-of-Cure Negative (n = 105)	*p*-Value ^1^
HSV	0 (0%)	1 (1.0%; 0–3.4%)	1.000
HHV-6	3 (19%; 0–41.1%)	10 (10%; 3.4–15.6%)	0.377
HPV	9 (56%; 28.9–83.7%)	75 (71%; 62.4–80.5%)	0.250
*M. genitalium*	0 (0%)	5 (4.8%; 0.2–9.4%)	1.000

HSV—herpes simplex virus; HHV-6—human herpes virus 6; HPV—human papillomavirus. ^1^ Fisher’s exact test was used to calculate the statistical significance.

**Table 3:** The *C. trachomatis ompA* genotype distribution in the urogenital samples of women who gave a *C. trachomatis* test-of-cure positive (n = 14) and a *C. trachomatis* test-of-cure negative (n = 94) sample. A test-of-cure sample was not available for 38 women.

Genotype	*C. trachomatis* Test-of-Cure Positive	*C. trachomatis* Test-of-Cure Negative	*p*-Value ^1^	No Test-of-Cure Sample Available	All Samples
E	8 (57%)	37 (39%)	0.251	24 (63%)	69 (47%)
F	3 (21%)	27 (29%)	0.753	9 (24%)	39 (27%)
G	2 (14%)	10 (11%)	0.653	1 (3%)	13 (9%)
K	0 (0%)	11 (12%)	0.353	0 (0%)	11 (8%)
D	0 (0%)	5 (5%)	1.000	3 (8%)	8 (5%)
H	1 (7%)	3 (3%)	0.431	0 (0%)	4 (3%)
J	0 (0%)	1 (1%)	1.000	1 (3%)	2 (1%)
Total	14 (100%)	94 (100%)		38 (100%)	146 (100%)

^1^ Fisher’s exact test was used to calculate the statistical significance.
